# Inconsistent Distances in Substitution Matrices can be Avoided by Properly Handling Hydrophobic Residues

**DOI:** 10.4137/ebo.s885

**Published:** 2008-10-09

**Authors:** J. Baussand, A. Carbone

**Affiliations:** 1 Génomique Analytique, Université Pierre et Marie Curie, INSERM U511, 91, Bd de l’Hôpital, 75013 Paris, France

**Keywords:** substitution matrices, triangle inequality, amino acids space, hydrophobic block

## Abstract

The adequacy of substitution matrices to model evolutionary relationships between amino acid sequences can be numerically evaluated by checking the mathematical property of triangle inequality for all triplets of residues. By converting substitution scores into distances, one can verify that a direct path between two amino acids is shorter than a path passing through a third amino acid in the amino acid space modeled by the matrix. If the triangle inequality is not verified, the intuition is that the evolutionary signal is not well modeled by the matrix, that the space is locally inconsistent and that the matrix construction was probably based on insufficient biological data. Previous analysis on several substitution matrices revealed that the number of triplets violating the triangle inequality increases with sequence divergence. Here, we compare matrices which are dedicated to the alignment of highly divergent proteins. The triangle inequality is tested on several classical substitution matrices as well as in a pair of “complementary” substitution matrices recording the evolutionary pressures inside and outside hydrophobic blocks in protein sequences. The analysis proves the crucial role of hydrophobic residues in substitution matrices dedicated to the alignment of distantly related proteins.

## Introduction

The study of persisting signals between homologous amino acid sequences allows for a better understanding of evolutionary processes in proteins. Due to specific evolutionary pressures, the rate of mutation and the type of substitution of sequence positions are strongly related to their functional and structural role. Evidence of differential evolution has been shown between residues belonging to either regular secondary structures or coil, and also between buried residues and exposed ones [Bowie et al. 1990; Bashford et al. 1987; Overington et al. 1992]. It has been observed that almost half of conserved positions between members of the same protein family are conserved in other protein families sharing the same fold [Friedberg and Margalit, 2001]. These conserved positions tend to be buried in the core of proteins and a significant fraction are located at critical positions for secondary structures. Those persistently conserved positions are mainly hydrophobic, which is consistent with the observation that protein structures are tolerant to residue substitutions preserving the hydropathic profile of the sequence [Krissinel, 2007].

Eigenvalue analysis of several substitution matrices associated to different levels of evolution reveals two modes of sequence conservation. Matrices constructed on homologous pairs of sequences with less than 30% identity are related to hydrophobicity and favor substitutions between residues of similar hydrophobic character. Matrices constructed on closer sequences are related to mutability of amino acids and act to disfavor any substitution [Kinjo and Nishikawa, 2004]. The transition point of these two modes of conservation in proteins revealed by substitution matrices is correlated with the twilight zone under which sequence alignment [Doolittle, 1981] and homology detection [Rost, 1999] become problematic. Thus, all these studies indicate that hydrophobicity is related to the structure of proteins and it seems to be one of the most conserved property when considering distantly related protein sequences.

Several substitution matrices have been developed in order to numerically evaluate the tendency of amino acids substitution along evolution and thus help to better identify equivalent positions between homologous proteins. These matrices are widely used to align sequences but their use to further understand the meaning of sequence evolution has also shown to be pertinent. The analysis of amino acid spaces modeled by substitution matrices can be realized by applying the known mathematical notion of triangle inequality to triplets of amino acids [Stojmirovic and Pestov, 2007]. Intuitively, the triangle inequality suggests that a direct path between two points in a space is never longer than a path passing through a third point. By converting substitution scores into distances, this property should hold for distances between triplets of amino acids in the space modeled by a substitution matrix. Verifying the number of amino acid triplets violating the triangle inequality allows for an evaluation of the quality of the evolutionary signal catched by substitution matrices. In fact, if the triangle inequality of an amino acid triplet fails, the intuition is that the evolutionary signal is not well modeled by the matrix and that the space is locally inconsistent. This might be due to missing information about intermediate mutations based on insufficient biological data.

The PAM [Dayhoff et al. 1978] and BLOSUM [Henickoff and Henickoff, 1992] series of substitution matrices have been proposed to align amino acid sequences accordingly to conservation levels among homologous sequences. The analysis of series of substitution matrices reveals that the number of triplets violating the triangle inequality increases with the divergence of sequences associated to the matrices [Stojmirovic and Pestov, 2007]. This result shows the difficulty to numerically model the evolutionary signal between divergent sequences and the importance of a rigorous methodology for the evaluation of substitution matrices modeling sequence evolution.

A pair of substitution matrices, IHBM (Inside Hydrophobic Blocks Matrix) and OHBM (Outside Hydrophobic Blocks Matrix), for amino acids sitting within and without hydrophobic blocks in sequences was constructed and successfully applied to the alignment of distantly related proteins [Baussand et al. 2007]. The notion of hydrophobic blocks, an unidimensional variant of the notion of hydrophobic clusters defined on sequences [Gaboriaud et al. 1987], detects closely located hydrophobic residues in a sequence and includes intercalating non hydrophobic ones if any. Hydrophobic blocks are correlated with regular secondary structures and buried regions, and so can be used as basic structural units to align protein sequences.

Here, we have tested the triangle inequality property on the hydrophobic blocks specific substitution matrices IHBM and OHBM as well as on the PAM and BLOSUM series and on the HSDM matrix, a distantly related sequences dedicated matrix. Two facts are highlighted. First, IHBM and OHBM matrices satisfy the triangle inequality constraints better than other matrices tested. This result underlines the relevance of considering the hydrophobic context when modeling sequence evolution and specifically divergent sequences. Secondly, all triplets of amino acids which do not satisfy the triangle inequality in the low sequence identity BLOSUM matrices (that is BLOSUM40 and BLOSUM30) involve hydrophobic residues. In other words, reasons for “inconsistency” of amino acid spaces modeled by substitution matrices dedicated to divergent sequences are due to hydrophobic residues. These two facts show that a particular care has to be taken for hydrophobic amino acids when constructing substitution matrices for distantly related proteins. This is in agreement with [Kinjo and Nishikawa, 2004; Pokarowski et al. 2007]. A global sequence approach cannot stand anymore when considering sequences under the twilight zone. The same conclusion is proposed in [Kinjo and Nishikawa, 2004] after an analysis of eigenvalues in matrices.

## Methods

### From a similarity measure to a distance measure

Let Ω be a set. A map *d*: Ω × Ω → ***R***^+^ is called a *quasi-metric* if it satisfies the properties: (i) for all *x*, *y* ε Ω , *d*(*y*, *x*) = *d*(*y*, *x*) = 0 ⇔ *x* = *y* and (ii) for all *x*, *y*, *z* ε Ω , *d*(*x*, *z*) ≤ *d*(*x*, *y*) + *d*(*y*, *z*) (triangle inequality). It is called a *metric* if in addition it satisfies *d*(*x*, *y*) = *d*(*y*, *x*) for all *x*, *y* ε Ω (symmetry).

Let *S*: Δ *×* Δ → *N* be a similarity score matrix, where Δ corresponds to the standard amino acids alphabet. The following properties hold: *S*(*x*, *x*) ≥ 0, *S*(*x*, *y*) = *S*(*y*, *x*), and *S*(*x*, *x*) ≥ *S*(*x*, *y*) for all *x*, *y* ε Δ . Substitution matrices can be investigated by transforming similarity measures between amino acids to *distance measures* by fixing *D*(*x*, *y*) = *S*(*x*, *x*) − *S*(*x*, *y*) [Stojmirovic and Pestov, 2007]. Since *S*(*x*, *x*) ≠ *S*(*y*, *y*) then *D*(*x*, *y*) ≠ *D*(*y*, *x*) and if the triangle inequality holds then *D* is a quasi-metric. Notice that *S* is defined with range *N* and this assures amino acid distances to take integer values. The matrices that we analyze are all integer matrices, but the approach holds for real matrices too.

### Triangle inequality and inconsistency

The triangle inequality is defined for a distance measure as *D*(*x*, *y*) ≤ *D*(*x*, *z*) + *D*(*z*, *y*) and it says that the shortest way to go from *x* to *y* in a quasi-metric space is the direct path. The quality of the evolutionary signal catched by a substitution matrix can be inferred in a quasi-metric space by verifying whether the distance *D* validates the triangle inequality property or not for all triplets of amino acids. If the triangle inequality is violated for *x*, *y*, *z* ε Δ , the distance (i.e. similarity) between *x*, *y* is thought not to represent evolution properly and we say that a “local inconsistency” takes place in the amino acids space modeled by the substitution matrix.

### Hydrophobic blocks definition

A significative periodic distribution of hydrophobic amino acids along protein sequences has been observed in [Callebaut et al. 1992] and periods seem to correlate to the regular secondary structures location in the folded protein: α-helices and β-sheets lying on the protein surface display contacts among hydrophobic residues at distance 3 or 4, and 2 respectively. Chains of amino acids lying within the hydrophobic core display consecutive hydrophobic residues, that is at distance 1. Based on this observation and on the fact that prolines can be seen as hydrophobic block breakers, a few simple combinatorial rules have been defined [Baussand et al. 2007] to detect *hydrophobic blocks* in sequences.

Hydrophobic blocks are correlated to regular secondary structures and buried regions, and are governed by different evolutionary pressures than regions outside hydrophobic blocks. A formal definition of hydrophobic blocks is given in [Baussand et al. 2007].

### Analyzed substitution matrices

#### PAM series

PAM matrices [Dayhoff et al. 1978] are conceived to compare pairs of sequences at *k* units distance (we speak about a PAM-*k* matrix). We say that two sequences *S*_1_ and *S*_2_ are at 1 PAM unit distance if *S*_1_ is converted into *S*_2_ with an average of 1 pointwise mutation per 100 residues. A PAM-*k* matrix is obtained by multiplying *k* times PAM-1 with itself and PAM-1 is calculated from global alignments of closely related proteins. Smaller the *k*, closer the evolutionary distance.

### BLOSUM series

BLOSUM matrices are derived from local, ungapped alignement (blocks) of closely or distantly related proteins [Henickoff and Henickoff, 1992]. All matrices of the series are calculated from suitable data sets of sequences by evaluating the log-ratio of observed and expected frequencies of pairs of amino acids. The smaller percentage of sequence identity for the blocks used to construct the matrix determines the number associated to the matrix. Greater the number, closer the evolutionary distance between blocks.

### HSDM matrix

The HSDM matrix is dedicated to divergent proteins. It is calculated on structurally equivalent positions (i.e. less than 5 Å) for pairs of proteins of less than 30% sequence identity [Prlic et al. 2000]. The HSDM matrix is originally a floating matrix and a discretized version is used here to allow for comparisons with other matrices.

### IHBM and OHBM matrices

Two complementary substitution matrices which have been constructed accordingly to hydrophobic blocks in homologous sequences [Baussand et al. 2007]: the IHBM matrix is specific of substitutions occurring within hydrophobic blocks and the OHBM matrix is specific of substitutions occurring outside hydrophobic blocks. The IHBM and OHBM matrices have been constructed on structurally equivalent positions (i.e. less than 5 Å) respectively inside and outside hydrophobic blocks from pairs of proteins of less than 30% sequence identity [Baussand et al. 2007].

### Evaluation of substitution matrices

We look for local inconsistencies in substitution matrices and use for this three different amino acid alphabets according to substitution matrix specificities. The alphabets are the complete alphabet *all* of 20 amino acids, the sub-alphabet α*_P_* of 19 amino-acids excluding proline and the sub-alphabet α*_Hy_* of 13 amino acids excluding the 7 hydrophobic amino acids used to define hydrophobic blocks (that is valine, leucine, isoleucine, methionine, phenylalanine, tyrosine, tryptophane).

For each matrix, we compute the distance *D*(*x*, *y*) = *S*(*x*, *x*) − *S*(*x*, *y*) for each pair of amino acids *x*, *y* within an alphabet, and verify the triangle inequality *D*(*x*, *y*) ≤ *D*(*x*, *z*) + *D*(*z*, *y*) for all triplets of amino acids *x*, *y*, *z* where *x* ≠ *z* and *y* ≠ *z* (notice that *x* can equal *y*). There are 7220 triplets for *all*, 6156 for α*_P_* and 1872 for α*_Hy_*. The number of failing triplets is the number of triplets for which *D*(*x*, *y*) ≤ *D*(*x*, *z*) + *D*(*z*, *y*). The *distance difference* between failing triplets is computed by *D*(*x*, *y*) − (*D*(*x*, *z*) + *D*(*z*, *y*)) and it measures the degree of violation : higher the distance difference greater the inconsistency. Notice that substitution scores are log-odd scores rounded to an integer, thus a distance difference of 1 can occur due to approximation.

## Results

### Analysis of classical substitution matrices

The triangle inequality has been verified for all triplets of amino acids for BLOSUM series, PAM series and HSDM substitution matrices (Table 1, *all* column). Results obtained for BLOSUM and PAM series agree with previous analysis [Stojmirovic and Pestov, 2007].

For PAM series, the number of failing triplets increases with a higher unit distance. The number of failing triplets is high even for PAM60 which corresponds to high level of sequence conservation. This indicates that many of the similarity scores in PAM60 do not model correctly evolutionary relationships between amino acids. All amino acids are involved in some failing triplets for PAM series with the exception of I, V and H for PAM60.

At the opposite, BLOSUM series present no failing triplets for matrices issued from pairs of sequences with more than 40% sequence identity. An exception occurs for BLOSUM70 which is probably due to round-off error as only 2 triplets involving the same three amino acids (A, I and V) fail, and their distance difference is 1. Failing triplets appear for BLOSUM40 and BLOSUM30 with a greater number of failing triplets and involved amino acids for BLOSUM30.

**Figure 1:**
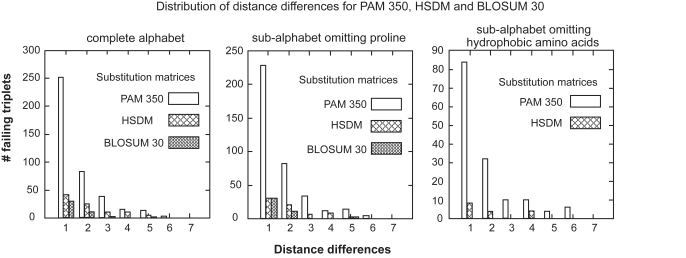
Distribution of distance differences for the three matrices dedicated to distantly related proteins: BLOSUM30, PAM350 and HSDM evaluated in the complete alphabet *All* (left), in the α*_P_* sub-alphabet (center) and in the α*_Hy_* sub-alphabet (right). Corresponding failing triplets are reported in Table 1.

Finally, the HSDM matrix presents twice as many failing triplets than BLOSUM30 with about the same number of involved amino acids : amino acids G, S, T are not involved in failing triplets for BLOSUM30, Q is not involved for HSDM. Distance differences for failing triplets vary between 1 and 5 for HSDM and BLOSUM30, and between 1 and 6 for PAM 350. A geometric-like distribution of distance differences for failing triplets in all three matrices dedicated to distantly related proteins is observed (see Fig. 1, left). The distribution shows that, although the majority of triangle inequality violations have distance differences equal to 1 for all matrices (this is possibly due to round-off effects), some important local inconsistencies of the amino acid space might occur.

### Analysis of substitution matrices derived from hydrophobic blocks

A different bias in amino acid composition is observed inside and outside hydrophobic blocks: prolines are excluded from hydrophobic blocks and hydrophobic amino acids tend to be excluded from regions outside hydrophobic blocks. The compositional bias, observed with respect to hydrophobic blocks in sequences, is reflected in IHBM and OHBM substitution matrices by a negative identity scores for amino acids which are excluded from the corresponding region. This implies that for some amino acids *x*, *y*, *S*(*x*, *x*) < 0 and *S*(*x*, *x*) < *S*(*x*, *y*). Thus the conversion of the similarity measure into a distance measure does not hold anymore for the complete alphabet and as a consequence, we verify the triangle inequality of amino acid triplets in the IHBM and OHBM matrices over two sub-alphabets which represent amino acid composition inside and outside hydrophobic blocks. Evaluation of the IHBM matrix is performed over the sub-alphabet α*_P_* which omits prolines, and the evaluation of the OHBM matrix is performed over the sub-alphabet α*_Hy_* which omits hydrophobic residues. BLOSUM series, PAM series and HSDM substitution matrices have also been evaluated for the two sub-alphabets (Table 1, second and third columns).

The omission of proline does not essentially modify the results obtained for BLOSUM series, PAM series and HSDM. Among matrices dedicated to divergent sequences, IHBM obtains the smallest number of triplets violating the triangle inequality with only 2 failing triplets compared to 42 for BLOSUM30, 374 for PAM350 et 66 for HSDM among the 6156 triplets evaluated in the α*_P_* sub-alphabet (Table 1, α*_P_* column). The two failing triplets observed in IHBM involve the same three amino acids (C, D, S) and the distance difference for these triplets is 1 indicating a minor violation or a round-off error.

Similarly, the omission of hydrophobic residues does not essentially modify results previously obtained for PAM series and HSDM with 16/1872 and 138/1872 failing triplets on α*_Hy_* compared to 90/7220 and 408/7220 on the complete alphabet for HSDM and PAM350 (see Table 1, α*_Hy_* column). On the opposite, no failing triplets are found for the BLOSUM series and OHBM on α*_Hy_* . This means that all 44 failing triplets for BLOSUM30 involve at least one hydrophobic amino acid and that all non-hydrophobic amino acid triplets do satisfy the triangle inequality. The same holds for BLOSUM40 and BLOSUM70.

The degree of violation of the triangle inequality observed for different matrices on α*_P_* and α*_Hy_* is comparable to the one observed on the complete alphabet. The distributions illustrated in Figure 1 (center and right) are geometric-like and display a non negligible number of triplets with distance difference greater than 1. This indicates that important local inconsistencies occur, for certain matrices, even in reduced amino acid spaces.

## Discussion

For this study, we used a simple mathematical idea to evaluate the adequacy of substitution matrices to model evolutionary relationships between amino acids. This numerical evaluation provides a method to compare substitution matrices by passing through their mathematical properties, and it is independent on sequence datasets which are generally used to compare substitution matrix performances. This point becomes especially important when highly divergent protein sequence datasets are tested for which reliable reference alignments are difficult to obtain.

### Amino acid space modeled by classical matrices

The high number of failing triplets and the important distance differences observed for matrices constructed on divergent sequences reveals the difficulty to well model amino acid space when considering distantly related proteins. The amino acid space modeled by PAM series is shown to be “inconsistent” with a high number of failing triplets even for matrices derived from close homologs. On the opposite, BLOSUM series are shown to better model amino acid substitutions according to the triangle inequality criteria as less failing triplets are observed. This suggests that BLOSUM matrices are more efficient than PAM matrices to align sequences in agreement with previous studies [Vogt et al. 1995; Prlic et al. 2000; Baussand et al. 2007].

Even though the HSDM matrix has been observed to have a better behavior than BLOSUM matrices for aligning distantly related proteins, HSDM presents a worst behavior towards the triangle inequality property than BLOSUM matrices. Reasons for this are unclear, but differences in their construction (BLOSUM uses conserved blocks of sequences and HSDM structurally equivalent positions, see Methods) might explain it. In this respect, notice that HSDM and IHBM and OHBM matrices are both constructed by using structurally equivalent positions for pairs of sequences of less than 30% sequence identity. The behavior of these matrices is consistent with our hypothesis: IHBM and OHBM matrices obtain less failing triplets and perform better for sequence alignment of distantly related proteins than HSDM [Baussand et al. 2007].

### Importance of the hydrophobic context

For the two sub-alphabets, all triplets of amino acids in IHBM satisfy the triangle inequality and only two triplets of amino acids involving the same three amino acids does not in OHBM. On the opposite, the number of failing triplets, relatively to the number of tested triplets and compared to the complete alphabet, remains almost unchanged for PAM series and HSDM in both sub-alphabets and for BLOSUM series in the sub-alphabet excluding prolines. Thus the evolutionary signal catched by the IHBM and OHBM pair of matrices satisfies the triangle inequality constraints better than classical matrices and induces better alignment of distantly related proteins [Baussand et al. 2007].

We observed that all failing triplets on the complete alphabet for the BLOSUM series involve hydrophobic amino acids and that all non-hydrophobic amino acid triplets satisfy the triangle inequality. Hydrophobic positions are very conserved along evolution but a high rate of mutation is observed between hydrophobic amino acids [Bowie et al. 1990]. Thus defining a distance measure on hydrophobic residues is expected to be difficult due to the information demanded on intermediate substitutions and to the difference of evolutionary pressures between hydrophobic and non-hydrophobic residues depending on their location. The observation on the BLOSUM series proves that hydrophobic amino acids play a crucial role in substitution matrices dedicated to distantly related proteins and that evolutionary pressures are then better modeled when the hydrophobic context of amino acids in proteins is explicitly considered as done for the IHBM and OHBM matrices.

Also, the fact that inconsistencies appear for PAM matrices dedicated to close homologs suggests that the construction method for PAM series might be not well adapted to model evolutionary pressures. In this respect, one cannot expect to avoid inconsistencies in PAM series by considering the α*_Hy_* sub-alphabet.

### Calculating substitution matrices with metric distances

In [Xu and Miranker, 2004a; Xu and Miranker, 2004b] a metric amino acids substitution matrix called mPAM has been derived by revisiting the mathematics used to derive PAM matrices as well as the original data [Dayhoff et al. 1978]. Values in the matrix satisfy the triangular inequality by construction and represent expected time between substitutions. The intuition behind the construction is that an amino acid pair with high substitution rate should take less time to appear than a pair with lower substitution rate. mPAM turns out to be more efficient than PAM matrices for homology search. This result confirms that matrices respecting metric properties, like the triangular inequality, better model amino acid substitutions. Revisiting the construction of substitution matrices by forcing the triangular inequality to hold and by explicitely taking into account the hydrophobic context in sequences should further improve the modelisation of the amino acid space associated to distantly related proteins.

## Figures and Tables

**Table 1:** Numbers of failing triplets for BLOSUM series, PAM series and HSDM substitution matrices for alphabets *all*, α*_P_* and α*_Hy_*. IHBM and OHBM evaluation is reported for *α_P_* and α*_Hy_* . The number of amino acids involved in the failing triplets is indicated in parenthesis. Last row: total number of evaluated triplets and total number of amino acids in the alphabet (in parenthesis). The symbol “–” indicates that the triangle inequality evaluation of the matrix is inadequate because of the alphabet.

Matrices	# Failing triplets					
	*All*		α*_P_*		α*_Hy_*	
BLOSUM90	0		0		0	
BLOSUM80	0		0		0	
BLOSUM70	2	(3)	2	(3)	0	
BLOSUM62	0		0		0	
BLOSUM60	0		0		0	
BLOSUM50	0		0		0	
BLOSUM40	6	(7)	6	(7)	0	
BLOSUM30	44	(17)	42	(16)	0	
PAM60	40	(17)	38	(16)	14	(9)
PAM120	92	(20)	72	(19)	46	(13)
PAM160	126	(20)	106	(19)	56	(13)
PAM250	178	(20)	158	(19)	60	(13)
PAM350	408	(20)	374	(19)	138	(13)
HSDM	90	(19)	66	(18)	16	(9)
IHBM	–		2	(3)	–	
OHBM	–		–		0	
Tot. # triplets	7220	(20)	6156	(19)	1872	(13)
